# A Multiplex PCR Assay for Simultaneous Detection of *Giardia duodenalis*, *Cryptosporidium parvum*, *Blastocystis* spp. and *Enterocytozoon bieneusi* in Goats

**DOI:** 10.3390/vetsci11090448

**Published:** 2024-09-22

**Authors:** Xingang Yu, Hui Xu, Xuanru Mu, Kaijian Yuan, Yilong Li, Nuo Xu, Qiaoyu Li, Wenjing Zeng, Shengfeng Chen, Yang Hong

**Affiliations:** 1 School of Life Science and Engineering, Foshan University, Foshan 528231, China; yuxingang4525@fosu.edu.cn (X.Y.); 2112359031@stu.fosu.edu.cn (H.X.); 2112209147@stu.fosu.edu.cn (X.M.); 2112209140@stu.fosu.edu.cn (K.Y.); 2112359111@stu.fosu.edu.cn (Y.L.); 20210420304@stu.fosu.edu.cn (N.X.); 2112459167@stu.fosu.edu.cn (Q.L.); 2112459159@stu.fosu.edu.cn (W.Z.); 2 National Institute of Parasitic Diseases, Chinese Center for Diseases Control and Prevention (Chinese Center for Tropical Diseases Research), Key Laboratory of Parasite and Vector Biology, National Health Commission of the People’s Republic of China (NHC) (Institute of Parasitic Disease Prevention and Control, Chinese Centre for Disease Control and Prevention, China), Shanghai 200025, China; 3 Hainan Tropical Disease Research Centre (Hainan Branch of the National Research Centre for Tropical Diseases), Haikou 571199, China

**Keywords:** multiplex PCR, *Giardia duodenalis*, *Cryptosporidium parvum*, *Enterocytozoon bieneusi*, *Blastocystis* spp.

## Abstract

**Simple Summary:**

Gastrointestinal parasitic infections are highly prevalent among goat populations, and field samples commonly display mixed infections. *Giardia duodenalis*, *Cryptosporidium parvum*, *Blastocystis* spp., and *Enterocytozoon bieneusi* are four common zoonotic intestinal parasites with similar symptoms, including diarrhea, vomiting, and dehydration. In this study, a multiplex PCR method was developed for the differential detection of these four zoonotic protozoans. Genomic DNA extracted from 130 goat stool samples obtained from four farms in Zhanjiang city, Guangdong Province, China, was tested with both the single-target PCRs and the multiplex PCR assay developed. The positive rates of *G. duodenalis*, *C. parvum*, *Blastocystis* spp., and *E. bieneusi* were 23.08% (30/130), 24.62% (32/130), 41.54% (54/130), and 12.31% (16/130), respectively. Furthermore, the detection results showed a high prevalence of mixed infections of goat parasites, predominantly involving two parasite species. The findings of the multiplex PCR were consistent with those of single-target PCRs. This multiplex PCR method showed high specificity, sensitivity, accuracy, and cost-effectiveness, and it was suitable for the rapid large-scale screening of goat stool samples for infections caused by *G. duodenalis*, *C. parvum*, *Blastocystis* spp., and *E. bieneusi*. It thus provided a reliable and efficient tool for animal health management and disease monitoring.

**Abstract:**

*Giardia duodenalis*, *Cryptosporidium parvum*, *Blastocystis* spp. and *Enterocytozoon bieneusi* are four common zoonotic parasites associated with severe diarrhea and enteric diseases. In this study, we developed a multiplex PCR assay for the simultaneous detection of these four zoonotic protozoans in goat stool samples and assessed its detection efficiency. Specific primers were designed from conserved gene sequences retrieved from GenBank, and the PCR conditions were optimized. Genomic DNA from 130 samples was subjected to both single-target PCR and multiplex PCR. The multiplex PCR assay successfully amplified specific gene fragments (*G. duodenalis*, 1400 bp; *C. parvum*, 755 bp; *Blastocystis* spp., 573 bp; *E. bieneusi*, 314 bp). The assay sensitivity was ≥10^2^ copies of pathogenic DNA clones with high specificity confirmed by negative results for other intestinal parasites. The detection rates were 23.08% (30/130) for *G. duodenalis*, 24.62% (32/130) for *C. parvum*, 41.54% (54/130) for *Blastocystis* spp., and 12.31% (16/130) for *E. bieneusi*, matching the single-target PCR results. The sensitivity and predictive values were 100.00%. This multiplex PCR provided a rapid, sensitive, specific, and cost-effective approach for detecting these four parasites. It also provided essential technical support for the rapid detection and epidemiological investigation of *G. duodenalis*, *C. parvum*, *Blastocystis* spp., and *E. bieneusi* infections in goat fecal samples.

## 1. Introduction

*Giardia duodenalis*, *Cryptosporidium parvum*, *Blastocystis* spp., and *Enterocytozoon bieneusi* are four common intestinal parasitic protozoans with wide host ranges, which include domestic animals, wildlife, companion animals and humans [1,2,3,4]. Since the 1970s, giardiasis, caused by *G. duodenalis*, has become widespread or has occurred in outbreaks throughout the world, and it is listed by the World Health Organization as one of the neglected diseases that harm human health [5]. It is estimated that more than 280 million cases of human *giardiasis* occur annually worldwide [6]. *C. parvum* is an important zoonotic protozoan that infects the small intestinal epithelial cells of both humans and animals, causing persistent diarrhea and contributing significantly to fatal cases in infants [7,8,9]. *Blastocystis* spp. are globally distributed single-celled intestinal parasites, characterized by their diverse and minute morphology, for which molecular biology analysis is the principal method of identification. Studies have shown that goats and sheep can carry the ST1, ST3, ST4, ST5, and ST6 genotypes of *Blastocystis* spp., which also pose potential public health risks [2]. *E. bieneusi* has a diminutive morphology and is globally distributed, infecting a wide range of hosts and displaying genetic diversity. Among the microsporidian species known to infect humans, zoonotic *E. bieneusi* stands out as the most widespread, targeting the gastrointestinal tract and causing diarrhea, particularly in immunocompromised individuals [10].

Goats are a grain-saving type of livestock that provide high quality meat, cashmere, and milk. Since the late 1980s, China has consistently been one of the countries with the largest goat populations [11]. With the continuous development of the goat-farming industry, there is an increasing emphasis on intensive and scientific farming practices. However, gastrointestinal parasitic infections in goats are still highly prevalent, posing a significant constraint on the healthy development of goat farming [12,13,14]. Among these pathogens, the zoonotic protozoan parasites such as *G. duodenalis*, *C. parvum*, *Blastocystis* spp., and *E. bieneusi*, not only cause significant production losses in goats but also pose a substantial threat to human health through the cysts or oocysts excreted in goat feces [2,15]. Because the oocysts or cysts of these pathogens can persist in the environment for a long time, they warrant adequate attention, and vigilance is crucial. Several studies have detected the oocysts or cysts of these above four protozoans in environmental water samples and sewage [16,17,18,19].

Traditional diagnostic protocols are typically time consuming and often lack sensitivity and specificity [20,21]. Multiplex PCR is a specialized composite PCR technique that allows the simultaneous detection of multiple pathogens within the same reaction system using multiple primer pairs. This technology is characterized by its convenience, simplicity, cost effectiveness, and higher efficiency and thus demonstrates significant clinical applicability [22,23]. Several multiplex PCR assays for the diagnosis of parasitic diseases in animal populations have been documented that identify and detect various species of parasites [24,25,26]. Tu et al. [24] developed a multiplex PCR assay for detecting blood parasites in goats, specifically targeting *Plasmodium caprae*, *Theileria luwenshuni*, and *Babesia* spp., which enables the identification of both single and mixed infections and is suitable for large-scale monitoring. However, there have been no reports of the simultaneous detection of *G. duodenalis*, *C. parvum*, *Blastocystis* spp., and *E. bieneusi* derived in goats using multiplex PCR methods.

Because *G. duodenalis*, *C. parvum*, *Blastocystis* spp., and *E. bieneusi* are small and frequently occur in mixed infections in goats, conventional PCR or nested PCR methods fail to meet the demand for comprehensive detection. Therefore, in this study, we established a multiplex PCR method for the simultaneous detection of these four zoonotic intestinal parasites, which provided a simple, rapid, and efficient diagnostic approach for the large-scale detection and epidemiological investigation of mixed infections in goat populations.

## 2. Materials and Methods

### 2.1. Clinical Samples and Parasite DNA

Between October and December 2022, 150 stool samples from Leizhou black goats were collected from four farms in Zhanjiang city, Guangdong Province, China [2]. These samples were from 64 reserve goats (aged 19–30 months), 66 adult goats (aged >30 months), and 20 stool samples from captive juvenile goats (0–3 months of age). Each fresh fecal sample was individually placed in a disposable plastic pouch labeled with the farm, goat age, and date of collection. The samples were promptly transported to the laboratory on ice and stored in a 2.50% (*w*/*v*) potassium dichromate solution at 4 °C until processing within one week. One hundred thirty goat fecal samples were used to evaluate the detection performance of the multiplex PCR method established in this study. Twenty fecal samples from juvenile goats, confirmed to be negative for these pathogens with single pathogen PCRs, were used as background controls for the sample testing.

The genomic DNAs of *G. duodenalis*, *C. parvum*, *Blastocystis* spp., and *E. bieneusi*, together with the control DNA samples of *Fasciola hepatica*, *Schistosoma japonicum*, *Echinococcus granulosus*, *Moniezia* and *Homalogaster paloniae*, were maintained in our laboratory.

### 2.2. Genomic DNA Extraction

Each fecal sample was washed with distilled water to eliminate any potassium dichromate residues before DNA extraction, as previously described [2]. Approximately 200 mg of each fecal sample was used for DNA extraction with the E.Z.N.A.^®^ Stool DNA Kit (Omega Bio-tek Inc., Norcross, GA, USA), following the manufacturer’s protocol. The fecal DNA samples were stored at −20 °C before PCR analysis.

### 2.3. Primer Design

The PCR primers were designed with Primer Premier 6.00 (PREMIER Biosoft, Canada) based on the genomic sequences of *G. duodenalis* (XM_001710026.2), *C. parvum* (XM_626998.1), *Blastocystis* spp. (AB071000.1), and *E. bieneusi* (KJ719492.1) published in the National Center for Biotechnology Information GenBank. The four sets of specific primers had similar annealing temperatures and were expected to circumvent the generation of secondary structures or primer dimers. The primers information are given in Table 1.

### 2.4. Construction of the Standard Plasmids

Using the genomic DNAs of *G. duodenalis*, *C. parvum*, *Blastocystis* spp., and *E. bieneusi* as the templates, respectively, the target fragments were amplified with singleplex PCR. Each amplicon was purified with the FastPure^®^ Gel DNA Extraction Mini Kit (DC301-01) (Vazyme, Nanjing, China) and then cloned into the pMD18-T vector (TaKaRa, Dalian, China). The recombinant plasmids were purified by Fastpure^®^ Plasmid Mini Kit (DC201-01) (Vazyme, Nanjing, China).

### 2.5. Singleplex PCR Assays

The singleplex PCR assay for *G. duodenalis*, *C. parvum*, *Blastocystis* spp., and *E. bieneusi* was conducted in a 12.5 μL reaction mixtures, each containing of 1 μL of genomic DNA, 6.3 μL of 2× Taq PCR Master Mix (Vazyme, Nanjing, China), 0.5 μL of each of specific primer (100 μmol/L), and 4.2 μL of nuclease-free water. The thermocycling program consisted of 95 °C for 5 min, 35 cycles of denaturation at 95 °C for 30 s, annealing at 54.8 °C for 30 s, and extension at 72 °C for 86 s, which was followed by a final extension step at 72 °C for 10 min. The amplification products were then stored at 4 °C.

### 2.6. Multiplex PCR Assay

The reaction parameters for the multiplex PCR were optimized based on the results of the simplex PCRs. The optimal conditions were determined by varying the annealing temperature (52–60 °C) and primer concentrations (20–100 μmol/L). The amplification products were visualized by electrophoresis on 2.0% (*w*/*v*) agarose gels with GoldView Nucleic Acid Stain (Phygene, Fuzhou, China) and viewed under the Azure Biosystems C200 Gel Imaging System (APExBIO, Houston, TX, USA).

The multiplex PCR assay was conducted in a total reaction volume of 30.0 μL, containing 15.0 μL of 2× Taq PCR Master Mix (Vazyme, Nanjing, China), 1 μL each of all primers (25 μmol/L), 1.5 μL of recombinant plasmid, and 1 μL of nuclease-free water. The multiplex PCR was performed under the following cycling conditions: initial denaturation at 95 °C for 5 min, 35 cycles of denaturation at 95 °C for 30 s, annealing at 55 °C for 30 s, and extension at 72 °C for 86 s, which was followed by a final extension step at 72 °C for 10 min.

### 2.7. Sensitivity and Specificity

The sensitivity of the multiplex PCR was determined with serial dilutions of the recombinant plasmid for each parasite in nuclease-free water. The concentrations of the four parasite recombinant plasmids were calculated and sequentially diluted to 10^6^, 10^5^, 10^4^, 10^3^, 10^2^, 10, and 1 copy/μL according to the formula below.

$$Plasmid\;copy\;number\;(\mathrm{copies}/\mu L)=\frac{concentration\;(\mathrm{ng}/\mu L)\times 6.023\times 10^{14}}{base\;number\times 660}$$

The DNA of *Moniezia expansa*, *Schistosoma japonicum*, *Fasciola hepatica*, *Homalogaster paloniae* and *Echinococcus granulosus* were used as templates, and the four standard plasmids and distilled water were used as the positive and negative controls for amplification to evaluate the specificity of the multiplex PCR assay.

### 2.8. Analysis of the Clinical Samples

To assess the diagnostic efficacy of the newly developed multiplex PCR method, 130 goats stool samples were analyzed. These samples were also analyzed at the same time with the corresponding singleplex PCR. The single-target PCR results were used as the reference standard with samples showing positive amplification products considered positive. The concordance between these two methods was then evaluated. The sensitivity, specificity, positive predictive value, and negative predictive value of the multiplex PCR method were calculated with the following formulae [27,28].

$$Sensitivity=\frac{\left(\#true\;positives-\#false\;negatives\right)}{\#true\;positives}$$

$$Specificity=\frac{\#true\;negatives}{\left(\#true\;negatives+\#false\;positives\right)}$$

$$Positive\;predictive\;value=\frac{\#true\;positives}{\left(\#true\;positives+\#false\;positives\right)}$$

$$Negative\;predictive\;value=\frac{\#true\;negatives}{\left(\#true\;negatives+\#false\;negatives\right)}$$

## 3. Results

### 3.1. Multiplex PCR Assay Results

**Figure 1:**
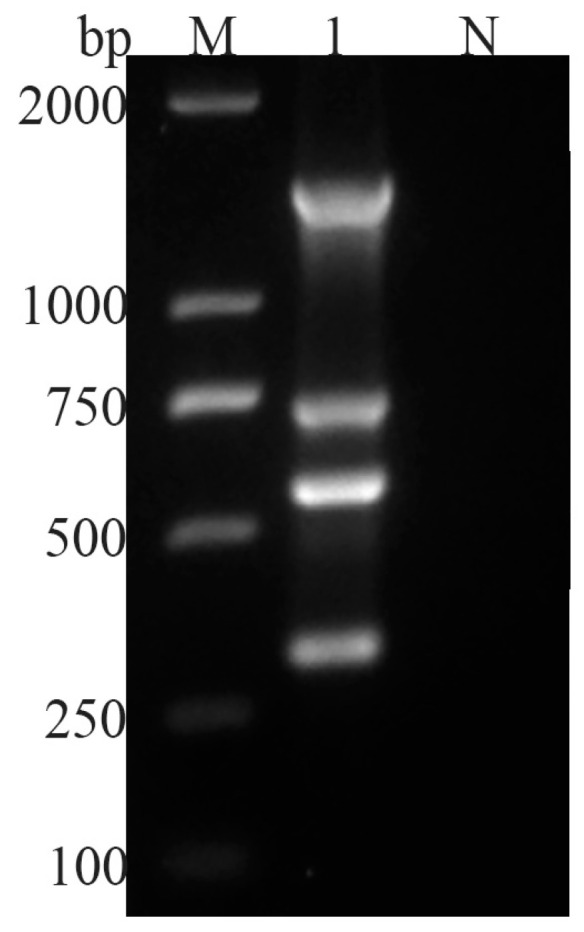
Multiple PCR detection of four parasites. M: 2000 bp DNA Maker, 1: four parasite PCR products, N: negative control.

The established multiplex PCR method simultaneously amplified the DNA of the four intestinal parasites in one tube, yielding specific bands for *G. duodenalis*, *C. parvum*, *Blastocystis* spp., and *E. bieneusi* with lengths of 1400 bp, 755 bp, 585 bp, and 314 bp, respectively (Figure 1).

### 3.2. Sensitivity and Specificity

**Figure 2:**
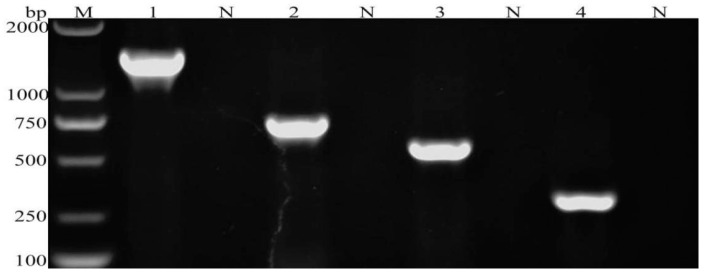
Multiple PCR detection of four parasites. M: 2000 bp DNA Maker, N: negative control, 1: *G. duodenalis*, 2: *C. parvum*, 3: *Blastocystis* spp., 4: *E. bieneusi*.

**Figure 3:**
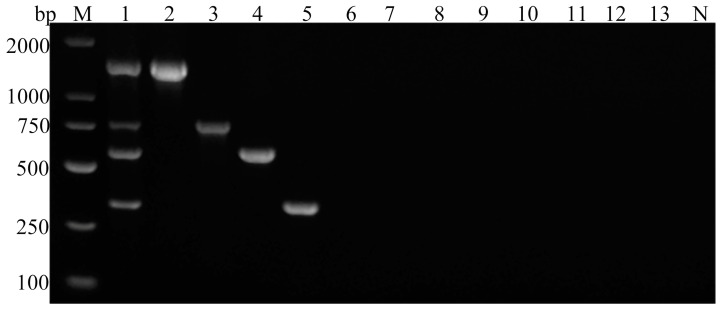
Specificity test of multiplex PCR. M: 2000 bp DNA marker, 1: *G. duodenalis*, *C. parvum*, *Blastocystis* spp., *E. bieneusi* plasmid mixture, 2: *E. bieneusi* DNA, 3: *Blastocystis* spp. DNA, 4: *C. parvum* DNA, 5: *G. duodenalis* DNA, 6: *E. granulosus* DNA, 7: *F. hepatica* DNA, 8: *S. japonicum* DNA, 9: *M. expansa* DNA, 10: *H. paloniae* DNA, 11: *Toxoplasma gondii* DNA, 12: *Eimeria arloingi* DNA, 13: *Neospora caninum* DNA, N: negative control.

The established multiplex PCR method successfully amplified four specific bands corresponding to the target genes of *G. duodenalis*, *C. parvum*, *Blastocystis* spp., and *E. bieneusi* (Figure 2). No cross-amplification was observed among these four parasitic species. Moreover, no amplification bands were detected for *F. hepatica*, *S. japonicum*, *E. granulosus*, *M. expansa* and *H. paloniae*, or sterile water (Figure 3).

**Figure 4:**
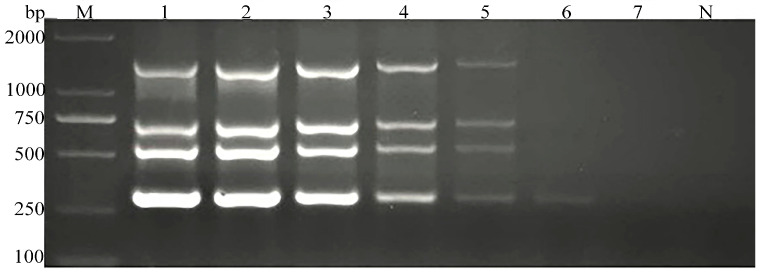
Sensitivity of multiplex PCR. M: 2000 bp DNA marker, 1: parasite DNA 1 × 10^6^ copies/μL, 2: parasite DNA 1 × 10^5^ copies/μL, 3: parasite DNA 1 × 10^4^ copies/μL, 4: parasite DNA 1 × 10^3^ copies/μL, 5: parasite DNA 10^2^ copies/μL, 6: parasite DNA 10 copies/μL, 7: parasite DNA 1 copy/μL, N: negative control.

The four standard plasmids were mixed together in a ratio of 1:1:1:1 into a single tube, diluted ten-fold serially at a concentration gradient of 10^0^ to 10^6^ copies/µL, and subsequently assessed to determine the assay’s sensitivity. The results showed detection limits of ≥10^2^ copies/μL for *G. duodenalis*, *C. parvum* and *Blastocystis* spp., and ≥10^1^ copies/μL for *E. bieneusi*, indicating the high sensitivity of the assay. Ultimately, the detection limit for all four parasitic DNA clones was determined to be ≥10^2^ copies/µL (Figure 4).

### 3.3. Field Evaluation of Multiplex PCR Assay

Of the 130 fecal samples tested, the positive rates for *G. duodenalis*, *C. parvum*, *Blastocystis spp*., and *E. bieneusi* were 23.08% (30/130), 24.62% (32/130), 41.54% (54/130), and 12.31% (16/130), respectively (Supplementary Tables S1 and S2).

Among the 80 positive stool samples detected, 11.25% (9/80) tested positive for only *G. duodenalis*, 11.25% (9/80) for only *C. parvum*, 31.25% (25/80) for only *Blastocystis* spp., and 2.50% (2/80) for only *E. bieneusi*. Overall, 27.50% (22/80) of the positive samples showed mixed infections with two parasite types. Specifically, 3.75% (3/80) were coinfected with *G. duodenalis* and *C. parvum*, 5.00% (2/80) with *G. duodenalis* and *Blastocystis* spp., 1.25% (1/80) with *G. duodenalis* and *E. bieneusi*, 11.25% (9/80) with *C. parvum* and *Blastocystis* spp., and 1.25% (1/80) with *C. parvum* and *E. bieneusi*. Furthermore, 5.00% (4/80) were coinfected with *E. bieneusi* and *Blastocystis* spp.

Furthermore, 11.25% (9/80) of the positive samples demonstrated mixed infections involving three parasite species. Specifically, 6.25% (5/80) were coinfected with *G. duodenalis*, *C. parvum*, and *Blastocystis* spp., 1.25% (1/80) with *G. duodenalis*, *C. parvum*, and *E. bieneusi*, and 3.75% (3/80) with *G. duodenalis*, *Blastocystis* spp., and *E. bieneusi*. Of the samples that tested positive for mixed infections, 5.00% (4/80) involved all four parasite species.

**Figure 5:**
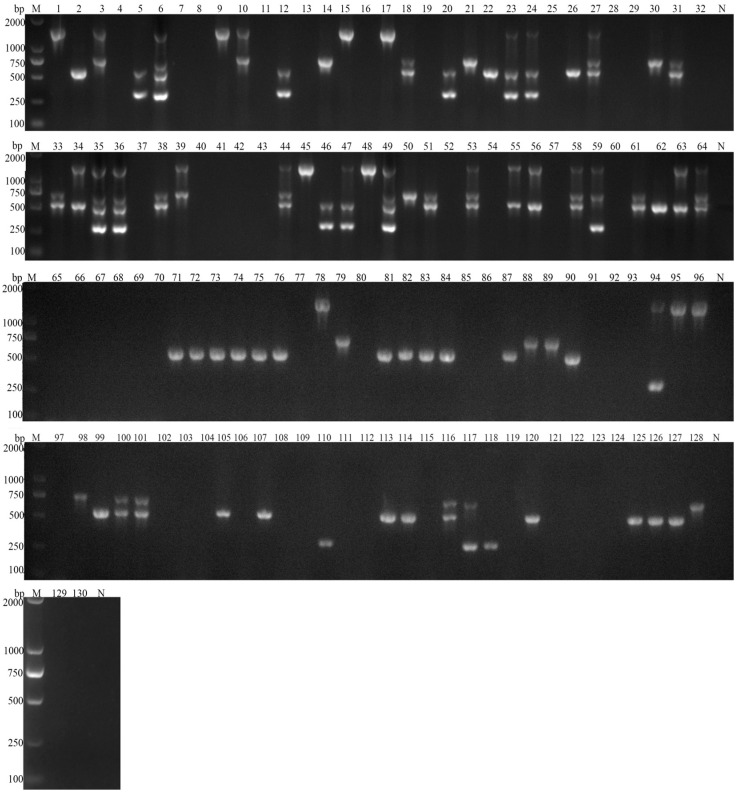
Multiplex PCR results of detecting black goat stool samples. M: 2000 bp DNA marker, 1–64: reserve goats DNA sample multiplex PCR test results, 65–130: adult goats DNA sample multiplex PCR, N: negative control.

These results indicated a high prevalence of mixed infections of parasites in goats with dual parasitic infections the predominant type of mixed infection observed (Figure 5).

### 3.4. Statistical Analysis

The results of the multiplex PCR were consistent with those of the single-target PCRs. Using the single-target PCR results as the gold standard, the multiplex PCR method demonstrated a sensitivity of 100.00% in detecting *G. duodenalis*, *C. parvum*, *Blastocystis* spp., and *E. bieneusi* DNA in goat fecal samples. Both the negative and positive predictive values were also 100.00% (Table 2).

## 4. Discussion

Gastrointestinal parasitic infections are highly prevalent in goat and sheep populations, and field samples commonly indicate mixed infections [2,29,30,31]. Wu et al. [31] surveyed the gastrointestinal parasitic infections in livestock in certain regions of the Tibet Autonomous Region of China. The results revealed an overall infection rate of 88.46% (230/260) in goats and 89.19% (553/620) in sheep with mixed infections of two to five parasite species accounting for 38.46% and 51.77% of infections, respectively. Peng et al. [32] investigated the infection rates of *G. duodenalis*, *Cryptosporidium* spp., and *E. bieneusi* in goats from multiple cities in Shaanxi and Henan Provinces of China. They found that 9.6% of the studied populations carried mixed infections of two pathogens, whereas 2.1% (13/629) of the animals were concurrently infected with all three pathogens. In a preliminary investigation [2], we examined the prevalence of parasitic infections in Leizhou black goats. Goats coinfected with two or three of *gastrointestinal protozoa* accounted for 11.50% (26/226) or 7.08% (16/226), respectively. Enteropathogenic zoonotic parasites not only cause significant production losses in goats due to various gastrointestinal diseases but also pose a potential public health threat [33]. Therefore, detecting and controlling these parasites are crucial for safeguarding public health and ensuring the well-being of animal populations worldwide.

Multiple molecular biology detection methods are currently available for the detection and identification of *Giardia duodenalis*, *C. parvum*, *E. bieneusi*, and *Blastocystis* spp. including single-pathogen nested PCR [34,35], recombinase polymerase amplification [36], loop-mediated isothermal amplification [37], high-resolution melting assay, and PCR–restriction fragment length polymorphism genotyping methods [38]. However, until now, there has been no study of the simultaneous detection of *G. duodenalis*, *C. parvum*, *Blastocystis* spp., and *E. bieneusi* with multiplex PCR methods.

During the development of multiple PCR detection methods, several key considerations are crucial, including primer design and the selection of the target gene sequence [23,39]. First, the primer design region must be highly specific to prevent the competitive amplification of different target fragments. The small subunit ribosomal RNA (*SSU rRNA*) gene is universally conserved across both eukaryotes and prokaryotes, evolving at a relatively constant rate. Regions within the gene that show greater sequence variability offer valuable insights into interspecies relationships [40,41]. *Blastocystis* spp. display significant morphological and genetic diversity. Therefore, the *SSU rRNA* gene is frequently used for molecular detection and genotyping of this organism [2,42]. The genus *Cryptosporidium* contains several distinct species. Differences in the rRNA gene at both the species and strain levels of *Cryptosporidium* allow researchers to accurately identify them in clinical and environmental samples [43]. Therefore, in the experiments reported here, we used both *Blastocystis* spp. and *Cryptosporidium SSU rRNA* as the target gene to detect these species. The *18S rRNA* gene is also highly conserved and is an ideal target gene for distinguishing parasite species and strains, and it is widely used in parasite taxonomy [44,45,46]. In this study, we selected the *18S rRNA* gene as the target gene for detecting *E. bieneusi*. Based on a sequence-specific alignment analysis, we previously identified a specific target gene sequence (XM_001710026.2) of *G. duodenalis* characterized by high stability and specificity. Second, to clearly differentiate the specific amplification products of the four pathogens, we ensured the generation of amplicons of significantly varying sizes based on the target genes: *G. duodenalis*, 1400 bp; *C. parvum*, 755 bp; *Blastocystis* spp., 573 bp; and *E. bieneusi*, 314 bp. The size disparities among the amplicons exceeded 150 bp, allowing the visual distinction of the bands of differing sizes on gel electrophoresis. Third, the annealing temperature is another critical factor influencing the specificity of the multiplex PCR assay. In this study, the annealing temperatures of the primers for each parasite were sufficiently similar to allow the successful amplification of all four fragments at the same temperature.

Specificity and sensitivity are critical indicators of the value of a multiplex PCR detection method, and they must be high to successfully confirm or exclude the presence of the target pathogens [25]. This multiplex PCR method successfully amplified target fragments of *G. duodenalis*, *C. parvum*, *Blastocystis* spp., and *E. bieneusi* of the expected sizes (Figure 1). No amplification of the DNA from other common intestinal parasites of goats, such as *Echinococcus granulosus*, *F. hepatica*, *S. japonicum*, *M. expansa*, or *H. paloniae*, was observed (Figure 3), demonstrating the high specificity of the multiplex PCR. In addition to the standard sterile water control, we incorporated juvenile goat feces as background controls in the experimental design to test the risk of nonspecific amplification from other related microorganisms. The sensitivity of a multiplex PCR is often influenced by several factors, such as the primer concentration, the number of primer pairs used, the DNA quality, the annealing temperature, and the cycle times [47,48]. In the initial phase of the experiment, bright bands appeared only for *G. duodenalis*, *Blastocystis* spp., and *E. bieneusi*, whereas *C. parvum* produced either no band or a weak band on agarose gel electrophoresis. After optimizing the PCR reaction components, we successfully addressed this problem by reducing the concentrations of the four primer pairs from 100 to 25 μmol/L. Furthermore, in clinical sample testing, although the results of multiplex PCR are consistent with those of simplex PCR, the positive bands of Cryptosporidium on agarose gel electrophoresis are not as pronounced as those observed in the simplex PCR reaction system. This suggests a potential trend of slightly decreased detection sensitivity in multiplex PCR, which is consistent with previous studies [24,46,49]. Competition between primers and the relatively limited amount of DNA templates in multiplex PCR often reduces the overall reaction sensitivity of the assay relative to that of the corresponding singleplex PCRs [22]. However, sensitivity testing indicated that the assay developed here achieved a limit of detection of ≥10^2^ copies/μL of the plasmids containing the cloned parasite DNA, indicating satisfactory sensitivity (Figure 4).

Subsequently, this study employed the singleplex PCRs to analyze 130 goat stool DNA samples, identifying 80 samples positive for parasite infections. Of these, single parasite infections accounted for 56.25% (45/80), whereas samples with mixed infections of two, three, and four parasites constituted 27.50% (22/80), 11.25% (9/80), and 5.00% (4/80), respectively. These results indicated a relatively high prevalence of mixed parasitic infections in goats, with dual-parasite infections predominating, which is consistent with our previous sequence alignment analysis [2]. These samples were also analyzed simultaneously with the multiplex PCR established in this study. The multiplex PCR results were consistent with those of the single-target PCRs. Using the single target PCR results as the reference standard, the multiplex PCR demonstrated a sensitivity of 100.00% in detecting *G. duodenalis*, *C. parvum*, *Blastocystis* spp., and *E. bieneusi* DNA in goat fecal samples. Both the negative and positive predictive values were also 100.00%.

Single pathogen PCR methods are limited to detecting individual pathogens, requiring additional diagnostic time and reagents when applied to each suspected infection sample. In contrast, multiplex PCR allows the simultaneous detection of multiple pathogens in a single test, facilitating a rapid diagnosis based on a gel electrophoretic analysis. Therefore, this approach not only reduced testing time but also conserved labor and material resources while facilitating rapid and accurate pathogen detection.

## 5. Conclusions

*Giardia duodenalis*, *C. parvum*, *Blastocystis* spp., and *E. bieneusi* are prevalent zoonotic intestinal parasites that not only cause significant production losses in goats but also pose a potential threat to public health. In this study, we successfully established a multiplex PCR detection method capable of simultaneously identifying these four parasites. The method showed high specificity, sensitivity, accuracy, and cost-effectiveness, and it was suitable for the rapid large-scale screening of goat stool samples for infections caused by these pathogens.

## Figures and Tables

**Table 1:** Primers used to detect *G. duodenalis*, *C. parvum*, *Blastocystis* spp., and *E. bieneusi* by multiplex PCR.

Parasite	Target (GenBank Accession)	Primer Sequence (5′-3′)	Fragment Size (bp)
*G. duodenalis*	*VSP with INR* (XM_001710026.2)	F: CCTGTGCTTAAGTTCCGACG	1400
		R: CGGTACTGCTAGCACATTCC	
*C. parvum*	*SSU rRNA* (XM_626998.1)	F: GCTCTTGGACCTTGGAAAACG	755
		R: GCTCGTCCATACCTTCAATCC	
*Blastocystis* spp.	*SSU rRNA* (AB071000.1)	F: GCCCTATCAGCTTTGGATGG	573
		R: GAATACCCCCAACTGTCCCT	
*E. bieneusi*	*18S rRNA* (KJ719492.1)	F: CAAGAGTGTCTATGGTGGATGC	314
		R: CGAACACTAAGATTTCCCCGC	

**Table 2:** Efficacy of the multiplex PCR assay for detection of four zoonotic protozoa infections in goat fecal samples.

Parasite	True Positive	False Negative	True Negative	False Positive	Sensitivity (%)	Specificity (%)	Positive Predictive Value (%)	Negative Predictive Value (%)
*G. duodenalis*	30	0	120	0	100	100	100	100
*E.bieneusi*	16	0	134	0	100	100	100	100
*C. parvum*	32	0	118	0	100	100	100	100
*Blastocystis* spp.	54	0	96	0	100	100	100	100

## Data Availability

The original contributions presented in the study are included in the article/supplementary material, further inquiries can be directed to the corresponding authors.

## Supplementary Materials

The following supporting information can be downloaded at https://www.mdpi.com/article/10.3390/vetsci11090448/s1, Table S1. Occurrence of *G. duodenalis*, *C. parvum*, *Blastocystis* spp., and *E. bieneusi* in black goats from four farms. Table S2. Distribution of *G. duodenalis*, *C. parvum*, *Blastocystis* spp. and *E. bieneusi* in 130 black goats.
