# Supplementary material for: A Multiplex PCR Assay for Simultaneous Detection of Giardia duodenalis, Cryptosporidium parvum, Blastocystis spp. and Enterocytozoon bieneusi in Goats
Source: Vet Sci. 2024 Sep 22;11(9):448. doi: 10.3390/vetsci11090448 (PMC11435618; doi:10.3390/vetsci11090448)
Supplement: Supplementary file 1 [file vetsci-11-00448-s001.zip › R1 Table S1. Occurrence of G. duodenalis, C. parvum, Blastocystis spp., and E. bieneusi in black goats from four farms..pdf]

Table S1. Occurrence of *G. duodenalis*, *C. parvum*, *Blastocystis* spp., and *E. bieneusi* in black goats from four farms.

Farm	Sample size	No. positive(%)	*G. duodenalis*	*C. parvum*	*Blastocystis* spp.	*E. bieneusi*
			No. positive(%)	No. positive(%)	No. positive(%)	No. positive(%)
Farm 1	47	32(68.09, 32/47)	10(21.28, 11/47)	20(42.55, 20/47)	20(42.55, 20/47)	5(10.64, 5/47)
Farm 2	34	21(61.76, 21/34)	3(8.82, 3/34)	4(11.76, 4/34)	17(50.00, 17/34)	1(2.94, 1/34)
Farm 3	29	16(55.17, 16/29)	9(31.03, 9/29)	6(20.69, 6/29)	10(34.48, 10/29)	2(6.90, 2/29)
Farm 4	20	11(55.00, 11/20)	8(40.00, 8/20)	2(10.00, 2/20)	7(35.00, 7/20)	8(40.00, 8/20)
Total	130		30(23.07, 30/130)	32(24.61, 32/130)	54(41.53, 54/130)	16(12.30, 16/130)
